# Supplementary figures and images for: A Retrospective Study of the Efficacy and Safety of Naldemedine for Treatment of Opioid-Induced Constipation in Patients with Hepatobiliary Pancreatic Cancer
Source: Medicina (Kaunas). 2023 Mar 2;59(3):492. doi: 10.3390/medicina59030492 (PMC10051263; doi:10.3390/medicina59030492)

## Supplementary Figure 1

Study period: June 2017 to August 2019

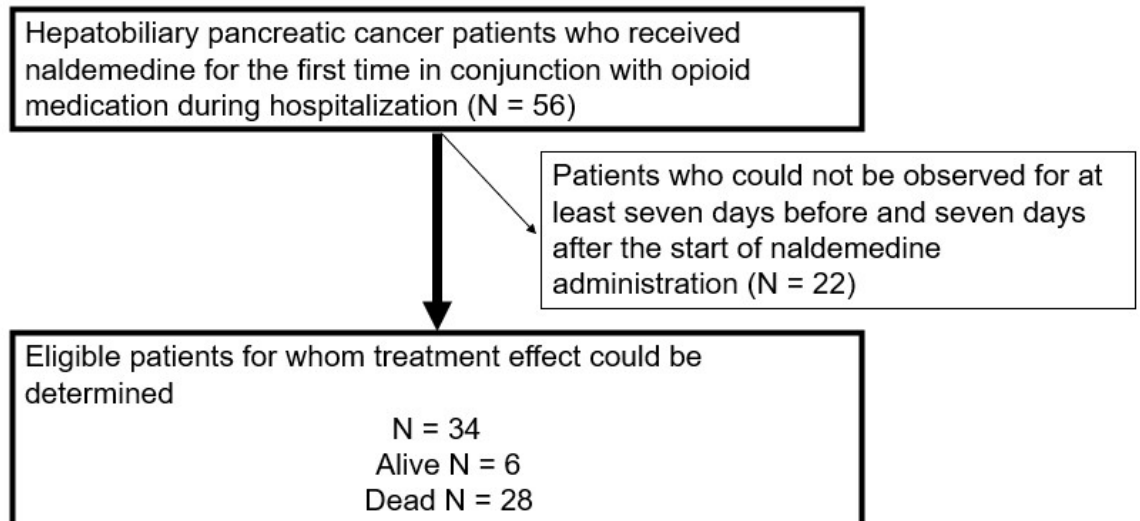

Data cutoff date  
September 30, 2019

Supplement: Supplementary file 1 [file medicina-59-00492-s001.zip › medicina-2115941-supplementary.pdf]
